# Coupling of mouse olfactory bulb projection neurons to fluctuating odour pulses

**DOI:** 10.1523/JNEUROSCI.1422-21.2022

**Published:** 2022-04-19

**Authors:** Debanjan Dasgupta, Tom P.A. Warner, Andrew Erskine, Andreas T. Schaefer

**Affiliations:** 1 Sensory circuits and Neurotechnology Laboratory, The Francis Crick Institute, London, United Kingdom; 2 Department of Neuroscience, Physiology & Pharmacology, University College London, London, United Kingdom

## Abstract

Odours are transported by turbulent air currents, creating complex temporal fluctuations in odour concentration that provide a potentially informative stimulus dimension. Recently, we have shown that mice are able to discriminate odour stimuli based on their temporal structure, indicating that information contained in the temporal structure of odour plumes can be extracted by the mouse olfactory system. Here, using *in vivo* extra- and intracellular electrophysiological recordings, we show that mitral and tufted cells (M/TCs) of the male C57BL/6 mouse olfactory bulb can encode the dominant temporal frequencies present in odour stimuli up to at least 20 Hz. A substantial population of cell-odour pairs showed significant coupling of their subthreshold membrane potential with the odour stimulus at both 2Hz (29/70) and the supra-sniff frequency 20Hz (24/70). Furthermore, M/TCs show differential coupling of their membrane potential to odour concentration fluctuations with tufted cells coupling more strongly for the 20Hz stimulation. Frequency coupling was always observed to be invariant to odour identity and M/TCs that coupled well to a mixture also coupled to at least one of the components of the mixture. Interestingly, pharmacological blocking of the inhibitory circuitry strongly modulated frequency coupling of cell-odour pairs at both 2Hz (10/15) and 20Hz (9/15). These results provide insight into how both cellular and circuit properties contribute to the encoding of temporal odour features in the mouse olfactory bulb.

## Introduction

Temporal structure has long been considered an integral part of sensory stimuli, notably in vision (Buracas et al., 1998; Kauffmann et al., 2014; Kauffmann et al., 2015; Borghuis et al., 2019; Chou et al., 2019; Wang et al., 2019) and audition (Nelken et al., 1999; Theunissen and Elie, 2014; VanRullen et al., 2014; Deneux et al., 2016). Odours in natural environments are transported by turbulent air streams, resulting in complex spatiotemporal odour distributions and rapid concentration fluctuations (Shraiman and Siggia, 2000; Celani et al., 2014; Pannunzi and Nowotny, 2019; Crimaldi et al., 2021; Marin et al., 2021). The neuronal circuitry of the olfactory system, particularly in invertebrates, has been shown to support the encoding of temporal structures present in odour stimuli (Hendrichs et al., 1994; Vickers and Baker, 1994; Vickers et al., 2001; Szyszka et al., 2014; Huston et al., 2015; Pannunzi and Nowotny, 2019). Temporal features in odour stimuli such as differences in stimulus onset were shown to be detectable on a behavioural level by bees (Szyszka et al., 2012; Sehdev et al., 2019). In mammals, recent reports have indicated that the neural circuitry of the early olfactory system readily sustains temporally modulated and precise action potential discharge (Cury and Uchida, 2010; Shusterman et al., 2011; Gupta et al., 2015) and is able to relay information about optogenetic stimuli with ~10 millisecond precision (Smear et al., 2011; Li et al., 2014; Rebello et al., 2014). Furthermore, we have recently shown that mice can utilise information in the temporal structure of odour stimuli at frequencies as high as 40 Hz to guide behavioural decisions (Ackels et al., 2021).

The olfactory bulb (OB) is the first stage of olfactory processing in the mammalian brain. Olfactory sensory neurons (OSNs) in the nasal epithelium convert volatile chemical signals into electrical activity forming the input to the OB. Air flow through the nasal cavity, transport of odours through the mucus, and the multi-step biochemical signal transduction together result in slow odour responses in OSNs (Sicard, 1986; Reisert and Matthews, 2001), creating the general notion that mammalian olfaction has a limited temporal bandwidth. While OSN activity indeed reflects a low pass filtered version of the incoming odour signal (Verhagen et al., 2007), information about different frequency components can still be present in OSN population activity (Nagel and Wilson, 2011). Moreover, circuit mechanisms in other brain regions and species have been shown to boost high-frequency content and sharpen stimulus presentation (Tramo et al., 2002; Atallah and Scanziani, 2009; Nagel et al., 2015; O'Sullivan et al., 2019). Given the intricate circuitry present in the OB, where multiple types of interneurons process incoming signals (Aungst et al., 2003; Fukunaga et al., 2012; Kato et al., 2013; Miyamichi et al., 2013; Fukunaga et al., 2014; Banerjee et al., 2015; Burton, 2017), we decided to investigate whether the OB circuitry plays a role in representing and processing the temporal features of odour stimuli.

Here, we show that mitral and tufted (M/T) cells, the OB output neurons, respond to odours - temporally modulated at frequencies of 2-20 Hz - in a frequency-dependent manner. Using whole-cell recordings, we show that subthreshold M/T cell activity *in vivo* can follow odour frequencies both at sniff and supra-sniff range for monomolecular odours and odour mixtures. We observe that while putative tufted (pTC) and mitral cells (pMC) show similar frequency coupling capacity at 2Hz, tufted cells have a higher propensity to follow odour frequencies at 20Hz. Pharmacologically "clamping" GABA receptors (Fukunaga et al., 2012) we show that local inhibition in the OB strongly modulates frequency coupling of M/T cells.

## Material and methods

### Experimental Design

All the experiments were performed using 5-8 weeks old C57BL6 male animals. For the unit recordings a Neuronexus poly3 probe was used for the whole-cell recordings standard borosilicate glass pipettes were used. This is described in detail below in their respective sections. The odour stimulation was performed using custom-built olfactory delivery device (tODD) run by custom written software in python. The details of the tODD are described in its respective section below.

### Animals

All animal procedures performed in this study were approved by the UK government (Home Office) and by Institutional Animal Welfare Ethical Review Panel. 5-8 weeks old C57/Bl6 males were used for the study. The study involved 6 animals for the extracellular unit recordings and 25 animals for the whole-cell patch recordings. The mice were housed up to 5 per cage under 12-12h light dark cycle with ad libitum food and water.

### Reagents

All odours were obtained at highest purity available from Sigma-Aldrich, St. Louis MO, USA. Unless otherwise specified, odours were diluted 1:5 with mineral oil in 15 ml glass vials (27162 (vials) & 27163 (screw-caps), Sigma-Aldrich).

### High-speed odour delivery device

A high speed odour delivery device was built as described previously (Ackels et al., 2021). Briefly, we connected 4 VHS valves (INKX0514750A, The Lee Company, Westbrook CT, USA) to odour containing 15ml glass vials (27162, Sigma-Aldrich) through individual output filters (INMX0350000A, The Lee Company). The vials were connected to a clean air supply (1L/min) through individual input flow controllers (AS1211F-M5-04, SMC Pneumatics). Each valve was controlled through a data acquisition module (National Instruments) controlled by a custom written script using Python software (PyPulse, PulseBoy; github.com/warnerwarner).

### In vivo electrophysiology

#### Surgical and experimental procedures

Prior to surgery all utilised surfaces and apparatus were sterilised with 1% trigene. 5-8 weeks old C57BL/6Jax mice were anaesthetised using a mixture of ketamine/xylazine (100mg/kg and 10mg/kg respectively) by injection inter-peritoneally (IP). Depth of anaesthesia was monitored throughout the procedure by testing the toe-pinch reflex. The fur over the skull and at the base of the neck was shaved away and the skin cleaned with 1% chlorhexidine scrub. Mice were then placed on a thermoregulator (DC Temperature Controller, FHC) heat pad which was controlled using feedback from a thermometer inserted rectally. While on the heat pad, the head of the animal was held in place with a set of ear bars. The scalp was incised and pulled away from the skull with 2 arterial clamps on each side of the incision. A custom head-fixation implant was attached to the base of the skull with medical super glue (Vetbond, 3M) such that its most anterior point rested approximately 0.5 mm posterior to the bregma line. Dental cement (Paladur, Heraeus Kulzer; Simplex Rapid Liquid, Associated Dental Products Ltd.) was then applied around the edges of the implant to ensure firm adhesion to the skull. A craniotomy over the right olfactory bulb (approximately ~2mm diameter) was made with a dental drill (Success 40, Osada) and then immersed in ACSF (NaCl (125 mM), KCl (5 mM), HEPES (10 mM), pH adjusted to 7.4 with NaOH, MgSO_4_.7H_2_O (2 mM), CaCl_2_.2H_2_O (2 mM), glucose (10 mM)) before removing the skull with forceps. The dura was then peeled off using a bent 30G needle tip.

Following surgery, mice were transferred to a custom head-fixation apparatus with a heat-pad (RS components) connected to a DC temperature controller (FHC) for recording. The animals were maintained at 37±0.5 °C.

#### Unit Recording

A Neuronexus poly3 probe was positioned above the OB craniotomy. An Ag/Ag^+^Cl^-^ reference coil was immersed in a well that was constructed of dental cement around the craniotomy. The reference wire was connected to both the ground and the reference of the amplifier board (RHD2132, Intan), which was connected (Omnetics) to a head-stage adapter (A32-OM32, Neuronexus). The probe, after zeroed at the OB surface, was advanced vertically into the dorsal OB at <4μm/s. This was continued until the deepest channels showed decrease in their recorded spikes indicating the end of the dorsal mitral cell layer. This was largely in the range of 400-600 μm from the brain surface. The signal from the probe was fed into a OpenEphys acquisition board (https://open-ephys.org/acquisition-system/eux9baf6a5s8tid06hk1mw5aafjdz1) and streamed through the accompanying GUI software (https://open-ephys.org/gui). The data was acquired at 30KHz and displayed both in a raw format, and a band pass filtered (300 - 6KHz) format. The band passed format was used primarily to visualise spikes across channels during the recording.

#### Odour stimulation (unit recordings)

Four odours (ethyl butyrate, 2-hexanone, isoamyl acetate, and eucalyptol) were diluted in mineral oil, as mentioned previously, in a ratio of 1:5.

**Fig 1:**
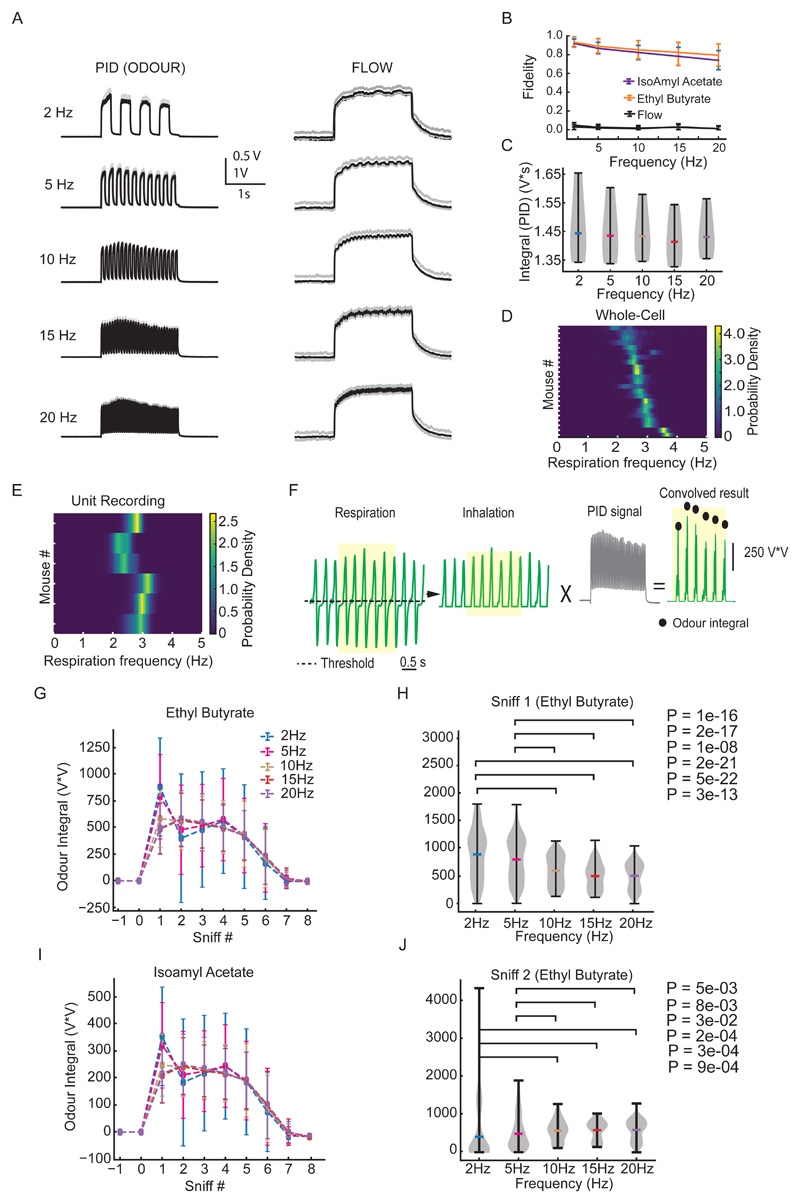
Respiration and odour interaction. **(A)** Photo-Ionisation Detector (PID) signal *(left)* and airflow *(right)* measurements of Ethyl Butyrate stimuli at 5 different frequencies. **(B)** Fidelity of the odour stimuli and flow at different frequencies. **(C)** Integral values of the total odour for the 5 frequencies, shown in (B), for 3 seconds after odour onset (2 seconds of odour and 1 second to allow full return to baseline). **(D)** Heatmap of the probability density of the respiration rate recorded from the animals during the whole-cell recordings (n=25). Each row represents an animal. **(E)** Same as (D) but from animals used for the unit recordings (n=6). **(F)** An example outlining the convolution method used to measure the odour signal present in single inhalations. The black dotted line represents the threshold for the start of inhalation. The black filled circles represent the odour integral obtained by convolving the inverted inhalation with the PID signal. **(G)** The odour integral in each inhalation for the entire duration of Ethyl Butyrate presentation. The filled circles represent mean values obtained from all the animals for a given frequency. The error bars represent the SD. **(H)** Violin plot of the odour integral values in the first sniff after the start of Ethyl Butyrate presentation. **(I)** Same as in (G) but for Isoamyl Acetate. **(J)** Same as in (H) but for sniff 2 for Ethyl Butyrate presentation.

Temporal structure of the odour stimulation was created using the VHS valves while blank valves helped maintain air flow to be constant through-out the stimulation period (Fig 1B). The start of a stimulation was always triggered to the start of inhalation which was continuously monitored online using a flow sensor (AWM2000, Honeywell). A minimum of 8s inter-trial interval was given for all the experiments.

The onset pulse was passed to the OpenEphys acquisition board so that the trial trigger was recorded simultaneously with the neural data. 800 total trials were presented during the experiment, consisting of 32 repeats of 5 different frequencies for 4 odours and 1 blank. Each trial lasted 2 seconds and was spaced by a minimum of 8 seconds between the offset of one trial and the onset of the following trial.

#### Whole-cell recording

Borosilicate pipettes (2x1.5 mm) were pulled and filled with (in mM) KMeSO_3_ (130), HEPES (10), KCl (7), ATP-Na_2_ (2), ATP-Mg (2), GTP-Na_x_ (0.5), EGTA (0.05) (pH = 7.3, osmolarity ~290 mOsm/kg). The OB surface was submerged with ACSF containing (in mM) NaCl (135), KCl (5.4), HEPES (5), MgCl_2_ (1), CaCl_2_ (1.8), (pH = 7.4 and ~300 mOsm/kg. Signals were amplified and low-pass filtered at 10 kHz using an Axoclamp 2B amplifier (Molecular Devices, USA) and digitized at 40 kHz using a Micro 1401 analogue to digital converter (Cambridge Electronic Design).

After zeroing the pipette tip position at the OB surface, we advanced the tip to reach a depth of ~ 200 μm from the surface. Next, we stepped at 2μm/s to hunt for a cell in a similar manner as described before (Margrie et al., 2002; Margrie and Schaefer, 2003; Jordan, 2021). Upon getting a successful hit, we released the positive pressure to achieve a gigaseal. The next gentle suction helped achieve the whole-cell configuration. We swiftly shifted to current-clamp mode to start a recording. Series resistance was compensated and monitored continuously during recording. Neurons showing series resistance >25 MΩ were discarded from further analysis.

The vertical depths of recorded neurons reported (e.g. Fig 4B & Fig 6E-F) are vertical distances from the brain surface. Respiration was recorded using a mass flow sensor (A3100, Honeywell) and digitized at 10KHz.

The GABA_A_ clamping experiments were performed as described before (Fukunaga et al., 2012). Briefly, muscimol and gabazine (Tocris Biosciences) were dissolved in ACSF to achieve a final concentration of 2mM (muscimol) and 0.4mM (gabazine). In a subset of experiments, this solution was superfused after ~10 minutes of recording under control conditions.

#### Odour stimulation (whole-cell recording)

Odours were presented as mixtures of monomolecular odorants mixed in 1:1 ratio which was eventually diluted in mineral oil in a 3:7 ratio. Odour A (ethyl butyrate + 2-hexanone) and B (isopentyl acetate + eucalyptol) were used for *in vivo* patch clamp experiments. Odour presentations were triggered on the onset of inhalation of the mouse as described for the unit recordings. The temporal structure of the odour pulses and the triggering of the blank valves were done as for the unit recording experiments described above. A minimum of 8s inter-trial interval was given for all the experiments.

### Analysis

#### Fidelity

Fidelity was defined here as the value of peak-to-trough of each square pulse normalised to the peak-to-baseline value. A fidelity of 1 therefore indicates that odour fully returns to baseline value between subsequent pulses, while a fidelity of ~ 0 for the flow indicates an almost continuous square pulse of air flow devoid of temporal structure.

#### Odour-Respiration Convolution

Photo-Ionization Device (PID) traces were taken of the odour stimuli from the same position as the mice were during the unit recording experiments. Each frequency was presented 4 times, with two different odours (ethyl Butyrate and isoamyl acetate) randomly presented with 10s of intertrial interval. This replicated the actual odour presentation to animals. The average signal from the 4 repetitions were used for the odour-sniff convolution outlined below.

##### Convolution step

Firstly, the respiration signal was high pass filtered, flipped, and median subtracted (such that the inhalation was now positive and exhalation negative). All values below 0 (and therefore any which were linked to the exhalation) were set to zero. Using the *find peaks* function, the times and heights of the inhalation peaks were recorded. The respiration trace was deemed to be in the inhalation phase when the signal had reached 5% of the total inhalation peak value and was deemed to have reached the end of inhalation when this threshold was reached again post peak.

The PID odour signal was resampled using *scipy.signal.resample* to have the same sampling frequency as the respiration signal (from 10kHz up 30kHz).

The inhalation only signal was convolved with the PID signal for the stimuli. Therefore, the resulting odour signal was modulated by the flow rate at any given time point. This convolved signal was then summed over the same time windows as in the binary convolution.

The convolutions were repeated for all presentations during the experiments using the same averaged PID signals. To compare the odour signal between all the frequencies, we applied Mann-Whitney U tests to the distributions of total odour calculated. The significance values for these tests were subjected to a Bonferroni correction to account for multiple comparisons.

#### Single Unit Responses

Unit responses to blank stimuli were first subtracted from the responses to odour stimuli at the same frequency. These subtracted responses were then averaged across repetitions of the same frequency to produce averaged subtracted cell responses (one per frequency). These responses were then z-scored so that they had an average response of 0 and a standard deviation of one, with each unit represented by an associated five value z-scored response vector. These z-scored responses were clustered using the *scipy.cluster.hierarchy.linkage* function, which groups units together by the distances between responses in the 5 dimensional space they occupy. The output was optimally ordered, ensuring that the global distances between neighboring cells was minimized.

#### pTC vs pMC classification

We classified our recordings (both unit and whole-cell) into putative tufted cells and putative mitral cells based on the methodology in Fukunaga et. al, 2012. We detected the exhalation peaks for every sniff cycle for the baseline period of a given recording which were then segregated into single sniffs with the corresponding spike-clipped membrane potential (whole- cell) or spike (unit recording). The membrane potential was then averaged over all sniff cycles while an average spike probability vector was created from the unit recording. Next, the sniff cycle and the thus obtained membrane potential or spike probability was converted into a phase plot, with phase 0 indicating peak exhalation. Then the preferred phase of the resultant membrane depolarisation or spiking probability were detected and considered 'the phase of respiration coupling' for the given cell/unit. Next, we classified cells into pTC if the phase of respiration coupling was in the range of 0-160° and pMC if in the range of 190-350°. Cells which did not have their phase in either of these ranges were not considered into any of the classes.

#### Spike sorting

**Fig 2:**
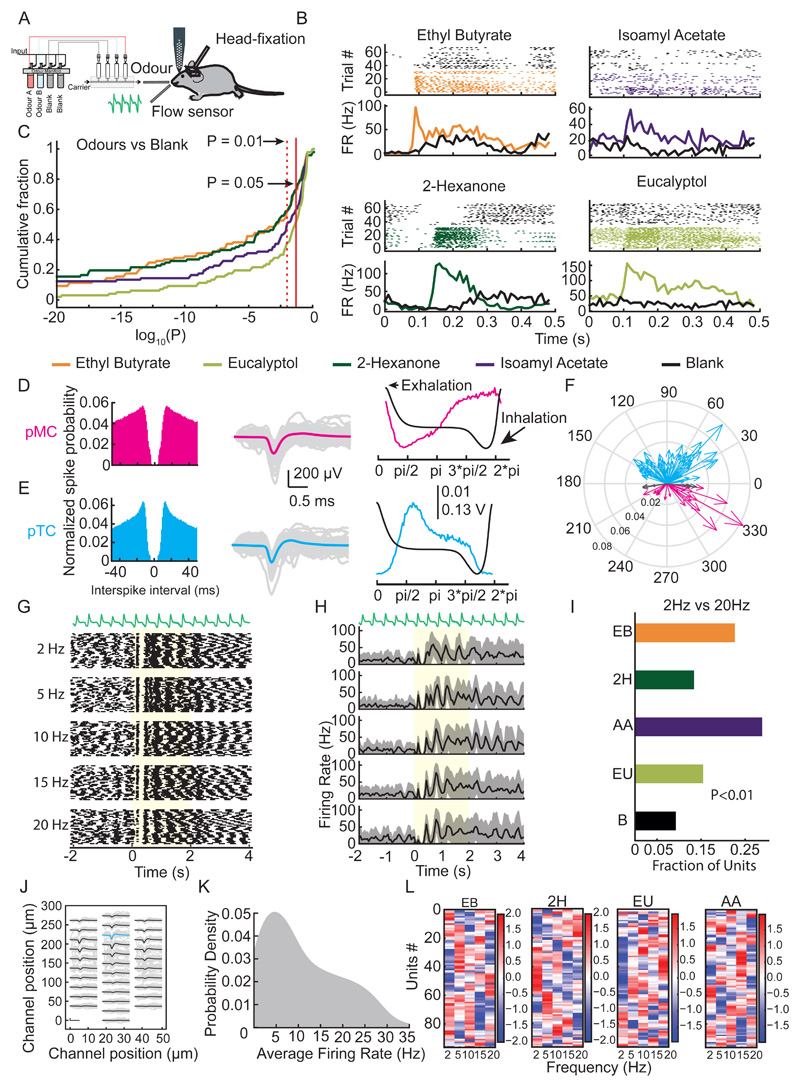
OB neurons encode odour frequency. **(A)** Schema of the unit recording experimental setup **(B)** Example recordings from single units for the odours and corresponding blank trials. For each subpanel, the top is the raster and the bottom is the average PSTH. **(C)** Cumulative fraction of units vs Log(P) values obtained by comparing the spike times in the first 500ms after odour onset with that for the blank trials. The P values were obtained using Mann-Whitney U test. **(D)** Autocorrelogram from a 'good' example putative mitral cell (pMC) ***(left),*** individual spike waveforms from the cell (grey) and its average waveform (magenta) ***(middle)*** and baseline spiking probability (magenta) overlaid on the respiration trace (black) ***(right).*** Peak spiking probability coincides with inhalation phase of the animal. **(E)** Same as in (D) but for putative tufted cell (cyan) with peak firing probability coincides with exhalation phase of the animal. **(F)** Summary Phase plot displaying peak firing probability of all recorded units against phase of respiration (n=64; pTC (cyan), n=26; pMC (magenta) and n=7; unresolved (black)). **(G)** Respiration trace **(top)** and raster plot for a single unit's response to five odour frequencies. **(H)** Respiration trace **(top)** and average PSTH of the same unit's responses in (G). **(I)** Fraction of units showing significant difference (P<0.01, Mann-Whitney U test) in firing rate in the first 500ms after odour onset between the 2Hz and 20Hz odour stimuli. The different colours represent the different odours used for all the units. (EB: Ethyl Butyrate; 2H: 2-Hexanone; AA: Isoamyl Acetate; EU: Eucalyptol; S: Shuffled). **(J)** Average waveform for the pTC in (E) across all channels of recording probe. Average waveforms shown in black, with the cyan average indicating the channel with the largest average waveform. **(K)** Baseline firing rate distribution of all recorded units across all experiments. **(L)** Z scored average change in spiking frequency in the first 500ms after odour onset compared to the baseline for ***(left to right)*** Ethyl butyrate, 2-Hexanone, Eucalyptol presentation and Isoamyl acetate presentation. The units have been sorted and grouped based on similarity in their activity for a given odour.

Kilosort2 (https://github.com/MouseLand/Kilosort2) was used to spikesort detected events into 'clusters'. Clusters were then manually curated using phy2 (https://github.com/cortex-lab/phy) and assigned a 'good', 'mua', or 'noise' label depending on if they were considered to be made up of neural spikes (good and multi-unit activity), or false detections (noise). The clusters made up of spikes were further divided into good or mua dependent on if they are thought to be spikes from a well isolated single unit or not. A 'good' unit is characterised by a well-defined rest period in its auto-correlogram, a characteristic spike waveform, and a stable firing rate and spike amplitude (however these can both vary throughout the recording) (Fig 2D-F). Only good clusters were used for further analysis.

#### Linear Classifier

97 clusters across 6 animals were grouped together and used for classification. Windows used to bin spikes varied both in size and in start relative to odour onset. Window starts span 0s to 3.99s from odour onset. The window sizes ranged from 10ms to 4s. All classifiers did not consider spikes from greater than 4s from odour onset. Therefore, a classifier with a 500ms window range could start between 0s and 3.5s, and a window range of 1s would use starts up to 3s from odour onset. This full range of starts and widths were used to determine both the time after odour onset that frequencies could be distinguished, and the time window required.

The data sets were always of dimensions 160 x 97 where 160 is the number of trials and 97 the number of clusters. The range of unit responses were scaled to have a mean response of 0 and a SD of 1. The data was split into a training (80%) and a test set (20%). The training set was used to train a linear Support Vector Machine (SVM) with a low regularisation parameter. The low regularisation parameter translates to less restrictions in weightings assigned to each cluster by the classifier. Once the classifiers had been trained, they were tested with the remaining 20% of trials. The trials to be saved for testing were picked at random. This training and testing were repeated 1000 times with a random selection of testing trials used each time. Classifiers were then trained on the same data but with their labels shuffled. To test how accuracy varied with number of clusters, random subsets of clusters were selected and used to train and test the classifiers.

Classifiers were then trained and tested on all one-to-one combinations of trials from the experimental data set. In this case a classifier was trained on all but two trials, one from each of the two trial types present in the training data. In this set chance was 50%. Finally, a series of classifiers were trained on all frequencies across all odours, with a single trial from every type being withheld for the test set. As there were 20 total trial types (5 frequencies with 4 odours) chance was 4%.

##### Single Cell Classifier

To test the accuracy of single cells in separating just 2Hz from 20Hz, a series of classifiers were trained and tested, in the method outlined above, on single cell responses to the 2Hz and 20Hz stimuli. These classifications were repeated 1000 times with different splits in the StratifiedKFold shuffle. The cells were then ordered from the lowest average classifier accuracy (across the four odours) to the highest classifier accuracy.

#### Change in membrane potential

The raw recordings were spike-clipped using a custom script in spike2 (Cambridge Electronic Design, UK). They were then stored into MATLAB (Mathworks, USA) readable files for further analysis.

All the recordings used were baseline-subtracted to rule out the effect of sniff related background membrane potential oscillations. This was done as described previously (Abraham et al., 2010). Briefly, stretches of baseline period were collated after matching the sniff-phase to that during the actual odour presentation. The membrane potential associated with these baseline periods was averaged to make a generic baseline trace for every cell. This was then subtracted from all the recorded traces during the odour-stimulation period to create a baseline subtracted trace.

For calculating the average change in membrane potential for 2 and 20Hz; we averaged the membrane potential in a 2s period pre-odour onset (Vm_base_). Next, we averaged the membrane potential in the first 500ms (~ 2 sniffs) post odour onset (Vm_odour500_) and subtracted from the baseline average voltage. In short;

$$\text{Avg}{.\;\text{change}\;\text{in}\;\text{membrane}\;\text{potential}\;=\;\text{Vm}}_{\text{odour500}}{\;-\;\text{Vm}}_{\text{base}}$$

#### Change in spike frequency

Action potentials were counted in the raw data and converted into spike frequency in bins of 50ms. Bar plot of the spike frequency yielded PSTH plots in Fig 4E. Further, we calculated the average spike frequency in 2s before onset (FR_base_) and 500ms post onset (FR_odour500_) and eventually subtracted them from each other to calculate the net change in spike frequency. In short;

$$\text{Avg}{.\;\text{change}\;\text{in}\;\text{spike}\;\text{frequency}\;=\;\text{FR}}_{\text{odour}500}{\;-\;\text{FR}}_{\text{base}}$$

#### Frequency-coupling coefficient estimation

Baseline-subtracted membrane potential traces for every odour and frequency were collected (Fig 5A *middle).* PID traces recorded for the 2Hz and 20Hz odour stimulation were averaged over 10 different trials (Fig 5A *top).* Next, we cross-correlated the PID trace and all the individual baseline-subtracted traces. This was repeated for all the trials for a given odour and frequency. We selected the peak correlation (CCodour_2Hz_ or CCodour_20Hz_) for all the trials. Similarly, we repeated the same exercise for the control blank stimulus which was also delivered at 2 and 20Hz and obtained a CCblank_2Hz_ or CCblank_20Hz_. Next, we normalized the CCodour with the CCblank for the respective frequencies and averaged them over all the trials to achieve a frequency-coupling coefficient (C*p*C) for a given cell-odour pair. In short for a given cell-odour pair:

$$\text{C}p\text{C}=\frac{{\text{CCodour}}_{2\text{Hz}}}{{\text{CCblank}}_{2\text{Hz}}}\;\text{and}\;CpC=\frac{{\text{CCodour}}_{20\text{Hz}}}{{\text{CCblank}}_{20\text{Hz}}}$$

#### Baseline control C*p*C

For every recorded cell, we isolated the baseline periods for all the trials. These were then baseline subtracted as described before. Next, we cross-correlated each of these baseline traces with the 2Hz and 20Hz PID signals to obtain the peak cross-correlation value. The C*p*C value for all the baseline traces for a given cell were then calculated in the same manner as described above. This set of C*p*C baseline 2Hz and 20Hz was then used to determine statistical difference from the C*p*C odour 2Hz and 20Hz.

#### Statistics

When only two groups were compared, a non-parametric Student's t test (paired and unpaired) have been used with Bonferroni correction used in the case of multiple comparison. When more than two groups were compared, we used one-way ANOVA. Bars and scatter plots are represented with mean ± SD of the population. The box plots are represented with the median (midline), with the 25^th^ percentile (top edge) and the 75^th^ percentile (bottom edge) while the minimum and the maximum values are represented by the top and bottom whiskers respectively. The violin plot in Fig 1C represents the distribution of all the datapoints with the midline representing the median value.

## Results

### M/T cells differentially respond to different frequencies in odour stimuli

We have previously shown that mice can behaviourally distinguish temporal structure in odours at frequencies up to 40 Hz (Ackels et al., 2021). Breathing in awake mice is highly variable vs almost metronomic in anaesthetized animals. Thus, in order to precisely probe the effect of temporal structure in odour stimuli on M/T cell activity, we recorded neural activity in anaesthetized mice, linking odour stimulation to the rhythmic breathing. We recorded extracellular spiking activity using Neuronexus silicone probes (97 units, 6 mice) from the dorsal OB while presenting 4 different odours (ethyl butyrate, 2-hexanone, amyl acetate and eucalyptol) at 5 different frequencies (2, 5, 10, 15 and 20Hz, Fig 1A-B) using a high-speed odour delivery device we recently developed (Ackels et al., 2021). As M/T cells can respond to changes in air pressure due to mechanosensitivity of OSNs (Grosmaitre et al., 2007), we offset changes in flow by presenting an odourless air stream from an additional valve following a temporal structure that operated anti-correlated to the odour valve. This resulted in approximately constant air flow profile throughout the odour presentation (Fig 1A-B). The temporal odour delivery device (tODD) allowed for a reliable odour pulse presentation (Fig 1B) with similar net volumes of odour for all the frequencies (P = 0.3142 Welch's t-test) (Fig 1C). To control for responses to residual flow changes, we included 'blank trials', i.e. trials identical to the odour trials in temporal structure, except that both the valves were connected to vials filled with mineral oil. Respiration was continuously monitored using a flow sensor placed in close proximity to the nostril contralateral to the recording hemisphere. Respiration frequency was 2.8 ± 0.5 Hz (mean ± SD, n=6 animals) (Fig 1E). To minimise sniff-cycle related variability (Shusterman et al., 2011) we triggered odour stimulation at the onset of inhalation. Further, we estimated the amount of odour that the animals might be inhaling during all the different frequencies. To do that we convolved the inhalation phase of every sniff cycle during the odour presentation with the recorded PID trace (Fig 1F). We then compared the thus convolved value for every sniff for the entire duration of a given odour presentation for all the different odour frequencies (Fig 1G&I). Next, we performed a pairwise statistical analysis between all the frequency combinations for a given sniff (Fig 1H&J). We observe that the odour integral during the first sniff varies marginally albeit significantly between the slow frequencies (2Hz & 5Hz) and the fast frequencies (10-20 Hz) (Fig 1H) while from the second sniff onwards there is no significant difference (Fig 1J). Further, we did not find any statistical significance between the slow 2Hz and 5Hz or among the fast 10Hz, 15Hz and 20Hz frequencies.

A typical recording session yielded recordings from multiple clusters from a depth of 300-500 μm from the OB surface. The recorded clusters were classified either as 'good' (well isolated clusters), 'MUA' (multi-unit activity, clusters which contained spikes of physiological origin but from numerous cells), or 'noise' (clusters containing spikes of non-physiological origin, e.g. electrical interference, movement artefacts) based on their autocorrelograms (Fig 2D-E *left),* waveforms (Fig 2D-E *middle),* firing rate and amplitude stabilities. Depending on the odour identity, between 49% and 72% of units displayed significant changes (P<0.05, Mann-Whitney U test) in their firing rates in response to the stimuli (Ethyl butyrate – 70/97; 2-Hexanone – 72/97; Amyl acetate – 56/97; Eucalyptol – 48/97) (Fig 2B-C). Corroborating previous findings (Fukunaga et al., 2012; Jordan et al., 2018b; Jordan et al., 2018a; Ackels et al., 2020) we observed that most of the units also coupled distinctly to either the inhalation (Fig 2D *right,* F) or exhalation phase (Fig 2E *right,* F) of the sniff cycle. From the entire population of recorded units, we estimated a total of 64 putative tufted cells (pTCs) and 26 putative mitral cells (pMCs) while 7 units could not be resolved into either of the class (Fig 2F). The average baseline firing of the recorded units was found to be 11 ± 9 Hz (mean ± SD) (Fig 2K).

Importantly, a subset of units displayed visibly different spiking profiles in response to different frequency odour stimuli (Fig 2G-H). Comparing activities of units between 2Hz and 20Hz stimuli we observed that a substantial number of units showed significant difference in their activity in the first 500ms after odour onset when compared to their response to the blank stimuli. This was true for all the 4 odours tested (Ethyl butyrate – 22/97; 2-Hexanone – 13/97; Amyl acetate – 28/97; Eucalyptol – 15/97; Blank – 9/97, P<0.01 Mann-Whitney U test) (Fig 2I). However, we did not find any obvious pattern between units responding for a given frequency among all the odours (Fig 2L).

**Fig 3:**
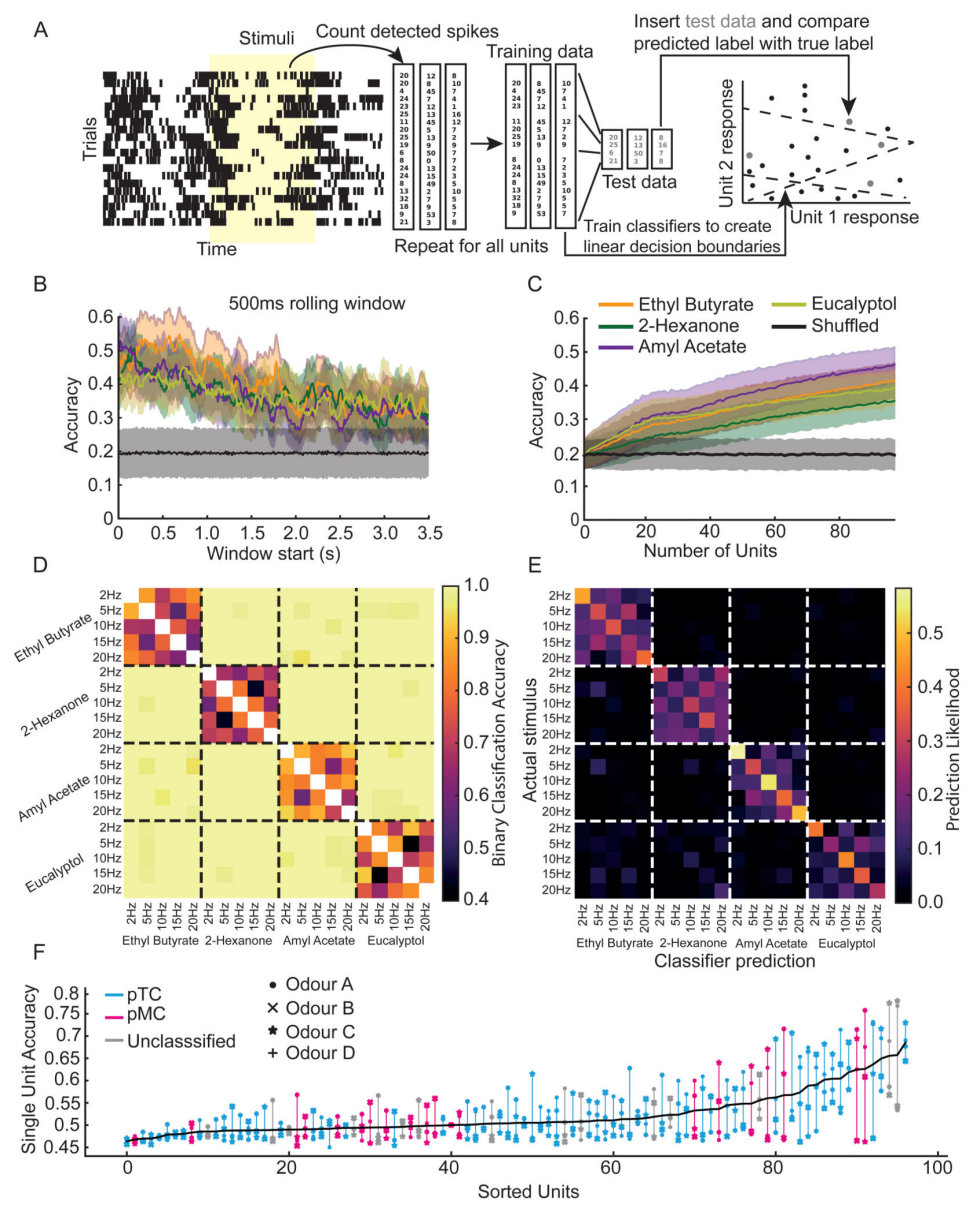
Classifiers to unit responses. **(A)** Schematic outlining of the procedure to train classifiers on unit spike times following an odour stimulus. **(B)** Average accuracy of linear SVM classifiers trained on a summed 500ms rolling window of detected unit spikes from different start times post odour onset. **(C)** Average accuracy of a set of linear SVM classifiers trained on the summed response from units 500ms from odour onset as the number of units available increases. **(D)** A matrix of all possible 1 vs. 1 comparisons between different trial types and the average accuracy of a linear SVM trained to distinguish between the two. Chance is 0.5 **(E)** A confusion matrix of linear fractional classifications for a set of classifiers trained to distinguish all trial types from one another. Y axis represents a trial's true label and the x axis represents a classifier's given label to a trial. Chance is 0.04. **(F)** The single cell responses to all four odours were used to train classifiers to distinguish responses to 2Hz and 20Hz. Each classifier only had access to a single unit's responses to a single odour. The accuracies from these classifiers were averaged across the four odours and used to sort the units. Each vertical line connects the accuracies to classifiers trained on the same unit but to different odours. Each odour is represented by a different symbol. The vertical lines and symbols are coloured by each unit's putative cell classification, cyan (pTCs), magenta (pMCs) or unknown (gray).

To examine the population level response to these different stimuli, we constructed response vectors by computing the cumulative spike count for all the 97 recorded units in the first 500ms post odour-onset and subtracting from it the spike counts of a blank trial. We trained linear classifiers on 80% of the data and tested on 20% to examine whether spiking activity obtained for different stimulus frequencies was linearly separable (Fig 3A). We observed that when we used a 500ms rolling window with temporal steps of 10ms, a classifier could achieve peak accuracies (Ethyl butyrate: 0.55 ± 0.08; 2-Hexanone: 0.51 ± 0.08; Amyl acetate: 0.53 ± 0.08; Eucalyptol: 0.45 ± 0.08) notably higher than what was obtained by training on shuffled data (0.2 ± 0.07) (Fig 3B). In addition, we trained classifiers on random subsets of unit responses, binned in a 500ms window from odour onset. The classifier accuracies increased with increasing number of units, with peak accuracies found for classifiers which had the full set of units available (Ethyl butyrate: 0.41 ± 0.05; 2-Hexanone: 0.35 ± 0.05; Amyl acetate: 0.46 ± 0.05; Eucalyptol: 0.39 ± 0.05) compared to shuffled data (0.2 ± 0.05) (Fig 3C). We then trained a series of classifiers to distinguish pairs selected from all possible combinations of odour frequencies and identities. We found that classifiers could readily distinguish responses for different stimulus frequencies well above chance (>0.5) for a given odour while performing even better while comparing responses for trials across different odours (Fig 3D). Next, we withheld one trial of each type of stimuli and trained a classifier with all the remaining trials. We tested the classifier on the withheld trials to see both how well it could distinguish trials, as well as explore the structure of the false classifications (Fig 3E). For a given odour, the predictability of a frequency of stimulus reached well above chance (> 0.04). Furthermore, when comparing across the different odours, the predictability was almost perfect (Fig 3E). Next, we trained classifiers based on the response of every unit to all the four odours and for the five different frequencies. Each of the classifier had access to a single unit's response for a single odour. The average accuracy obtained from all the odours for a given unit were sorted and plotted (Fig 3F). We could observe that both the pTC and pMC appeared at different accuracy values indicating that both the cell population responses were required to achieve the overall final accuracy values for the population. One should note, however, that the number of pTCs recorded were much larger than the number of pMCs.

Overall, this suggests that OB neurons can encode temporal structure present in odour stimuli at frequencies of at least up to 20Hz in their spiking pattern. Importantly, this indicates that information can be read out by downstream structures simply by summing activity over populations of M/TCs at relatively low temporal resolution (i.e. spike counts over 500 ms).

### M/T cells follow odour stimulus both at 2 and 20Hz

**Fig 4:**
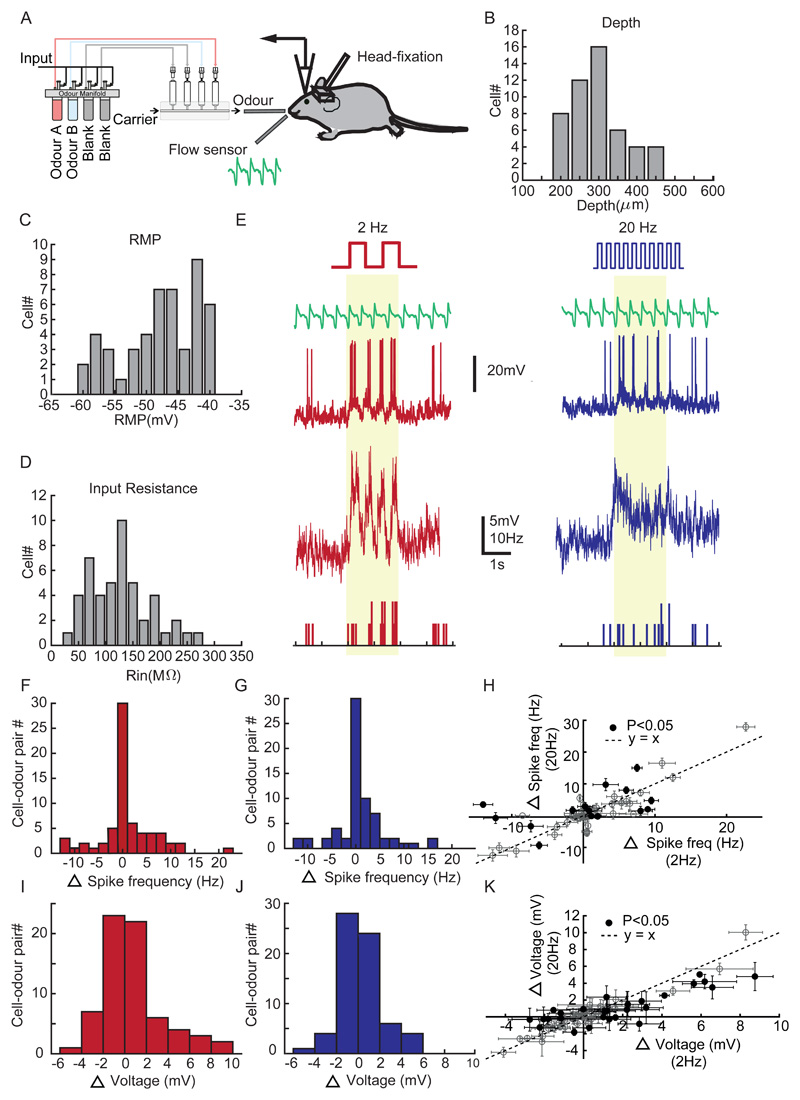
OB neurons respond to both 2 Hz and 20Hz temporally structured odour stimuli. **(A)** Schema of the whole-cell recording experimental setup. **(B)** Histogram representing the distribution of depth from the OB surface of all the recorded neurons, **(C)** resting membrane potential and **(D)** input resistance (n=42 cells, 25 mice) **(E)** Example recordings for 2Hz ***(left)*** and 20Hz ***(right)*** stimuli. From top to bottom, schema of odour stimulus, example respiration trace, example recording from a neuron, extracted V_m_ and PSTH. **(F)** Histogram of change in action potential frequency in the first 500ms compared to the baseline for 2Hz stimuli and **(G)** 20Hz stimuli. **(H)** Spike frequency change for 2Hz vs 20Hz for all cell-odour pairs. The hollow circles represent cell-odour pairs which showed statistically insignificant difference between the 2Hz and the 20Hz trials while the solid markers (15/70) represent cell-odour pairs showing significant difference (P<0.05, Two-tailed unpaired t-test). Each marker represents a cell-odour pair and the error bars represent the SD obtained from all the trials. **(I)** Histogram of change in average V_m_ in the first 500ms compared to the baseline for 2Hz stimuli and **(J)** 20Hz stimuli. **(K)** Avg. Change in V_m_ for 2Hz vs. 20Hz. The markers and error bars have similar meaning as in (H) but for change in V_m_ from baseline. 27/70 cell odour pairs showed significant difference between the 2Hz and 20Hz trials (P<0.05, Two-tailed unpaired t-test).

To better understand the mechanism that gives rise to frequency dependent M/T cell spiking responses and to get insight into their subthreshold basis, we performed whole-cell recordings from M/T cells (Fig 4A). To increase the probability of finding a responsive cell-odour pair and due to the time limitation and lower yield of stable whole-cell recordings, we employed odour mixtures as stimuli (A: Ethyl butyrate and 2-Hexanone; B: Amyl acetate and Eucalyptol) and presented odours at only two frequencies, 2Hz and 20Hz (Fig 4E). As with the unit recordings, stimuli were triggered at the onset of inhalation and blank trials were included. The animals had an average respiration frequency of 2.9 ± 1.3 Hz (mean ± SD, n=25 mice) (Fig 1D). We recorded from 42 neurons in 25 mice at depths of 180-450 μm from the surface of the olfactory bulb (Fig 4B). The neurons showed resting membrane potentials (RMP) ranging from -38 mV to -60 mV (Fig 4C) and input resistance of 45-280 MΩ (Fig 4D). These values are congruent with previous findings indicating that our recordings were largely from M/T cells (Margrie et al., 2001; Margrie et al., 2002; Margrie and Schaefer, 2003; Fukunaga et al., 2012; Jordan et al., 2018b; Jordan et al., 2018a). In response to 2 Hz odour presentation 25/70 cell-odour pairs showed significant changes in action potential discharge compared to the blank stimulus at the same frequency in the first 500ms after odour onset (13/35 for Mixture A, 12/35 for Mixture B). Of these, 14/25 cell-odour pairs significantly increased their firing in response to odour stimulation while 11/25 showed a significant decrease. To assess the subthreshold response, we calculated the average change in membrane potential (Δ Voltage) during the first 500ms of the stimulus from the baseline. With 2 Hz odour presentation 34/70 cell-odour pairs showed significant subthreshold responses compared to the blank stimulus at 2Hz (24/34 for Mixture A, 10/34 for Mixture B). Of these, 18/34 cell-odour pairs significantly depolarised and 16/34 hyperpolarised in response to odour stimulation. When presented with the 20 Hz odour stimulation, 18/70 cell-odour pairs showed significant spiking responses compared to the blank stimulus at 20Hz (12/18 for Mixture A, 6/18 for Mixture B). Of these, 11/18 cell-odour pairs significantly increased their firing in response to odour stimulation whereas 7/18 showed a significant decrease. On the subthreshold level, 25/70 cell-odour pairs showed significant odour responses to the 20 Hz odour stimulation (18/25 for Mixture A, 7/25 for Mixture B). Of these, 15/25 cell-odour pairs significantly depolarised in response to the odour stimulation and 10/25 hyperpolarised.

Comparing average change in action potential firing frequency in the first 500ms after odour onset from the baseline between the 2Hz and 20Hz responses (Fig 4F-H) we observed that for 15/70 cell-odour pairs, responses differed significantly between the two cases (Fig 4H, P<0.05, Two-tailed unpaired t-test). For 6/15 cell-odour pairs responses were significantly larger for the 2 Hz case. This is consistent with our findings from the unit recordings (Fig 2-3). Interestingly, a larger number of cell-odour pairs (27/70) showed significant differences between the subthreshold responses to the two stimuli (Fig 4I&K, P<0.05; Two-tailed unpaired t-test) compared to the suprathreshold response (18/27 cell-odour pairs showed significantly larger Δ Voltage responses for the 2Hz stimulus).

**Fig 5:**
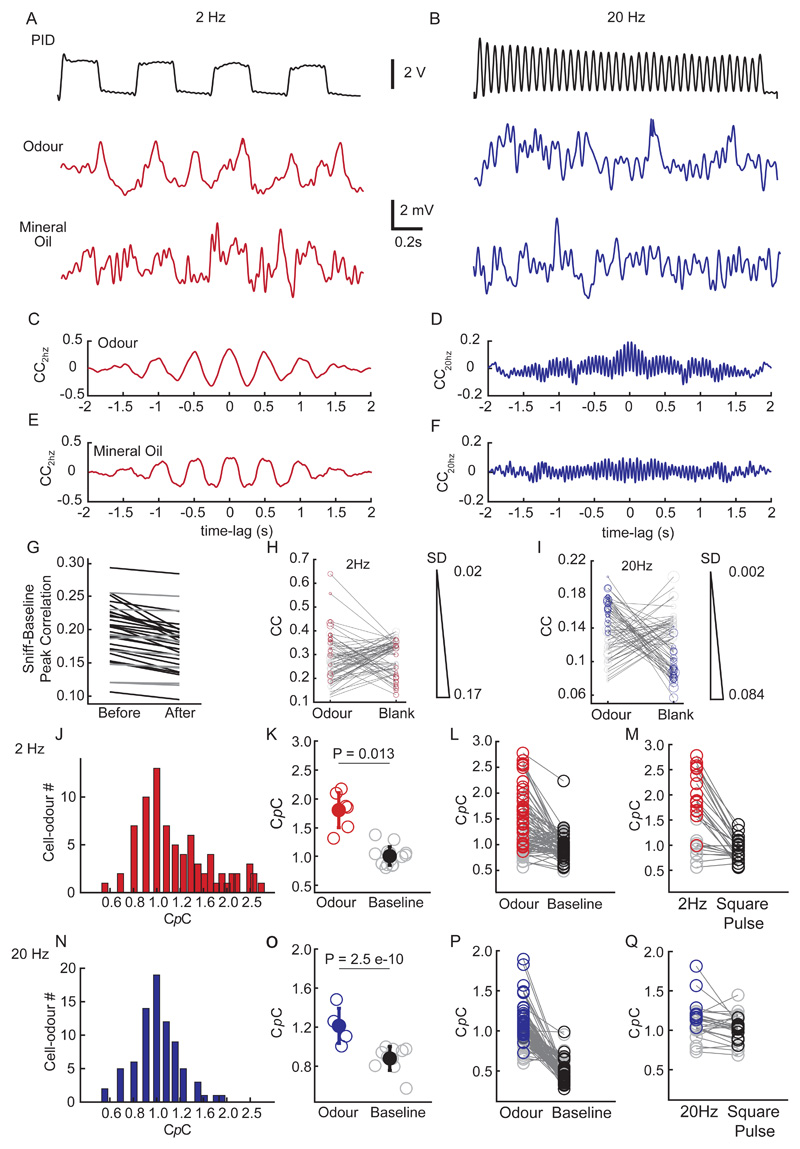
Olfactory bulb neurons can follow temporally structured odour stimuli. **(A)** (*Top*) An example trace recorded using a PID of a 2Hz odour stimulus; ***(Middle)*** an example V_m_ trace (action potentials clipped and baseline subtracted) recorded during 2Hz odour stimulus presentation; ***(Bottom)*** recording from the same neuron during 2Hz mineral oil stimulus presentation. **(B)** Same as in (A) but for a 20Hz stimulus from a different neuron. **(C)** Cross-correlation plot between 2Hz PID trace and 2Hz V_m_ odour trace; **(D)** 20Hz PID trace and 20Hz V_m_ odour trace; **(E)** 2Hz PID trace and 2Hz V_m_ mineral oil trace and **(F)** 20Hz PID trace and 20Hz V_m_ mineral oil trace. **(G)** Mean Peak correlation coefficient of the baseline period with the respective respiration stretches (2s) plotted as before and after the subtraction operation. Black lines denote cells showing significant decrease in correlation after the correction (34/42, P<0.05, Paired t Test). **(H)** CC_odour_ and CC_blank_ in cell-odour pair basis for the 2Hz case. Red open circles mark the cell-odour pairs which showed significant difference compared to the blank trials (n=20/70) (P<0.05, 2 Tailed t test). **(I)** Same as in (H) but for the 20Hz case. Blue open circles mark the cell-odour pairs which showed significant difference compared to the blank trials (n=16/70) (P<0.05, 2 Tailed t test). The marker size denotes the SD obtained from all the trials for a given cell-odour pair. **(J)** Histogram of coupling co-efficient (C*p*C) for all cell-odour pairs for 2Hz responses (n=70 cell-odour pairs, 25 mice. **(K)** C*p*C estimated from the individual odour trials (red hollow) and that from the baseline control (grey) of 2Hz odour stimuli for a given cell-odour pair. The filled circle represents the population average of C*p*C obtained from the odour trials (red solid) and the baseline controls (black) (P=0.013, Unpaired t test). **(L)** Odour C*p*C vs baseline C*p*C from all recorded cell-odour pairs. The grey markers (n=41/70) represent the cell-odour pairs showing non-significant difference between the two while the coloured markers (29/70) represent the significantly different cell-odour pairs. Significance threshold was set at P<0.05. **(M)** Estimated C*p*C for a subset of cell-odour pairs which were provided both the temporal stimulus and square pulse. Note the significant decrease in C*p*C for the square pulse compared to the 2Hz case. The red coloured open circles denote cell-odour pairs for 2Hz which showed significant difference from their paired square pulse trials (black open circles) (16/27 cell-odour pairs) (P<0.05; Two-tailed paired t test). **(N)** Same as in (J) but for 20Hz responses **(O)** Same as in (K) but for 20Hz responses. (n=24/70, significant cell-odour pairs) **(P)** Same as in (L) but for 20Hz responses. **(Q)** Same as in (M) but for the 20Hz case. The blue coloured open circles denote cell-odour pairs for 20Hz which showed significant difference from their paired square pulse trials (black open circles) (13/27 cell-odour pairs) (P<0.05; Two-tailed paired t test).

To quantify the coupling of membrane potential to the frequency of odour stimulation, we estimated the peak correlation-coefficient of the odour period (CC_odour_) and compared with that for the blank condition (CC_blank_) (Fig 5H-I). We observed that 20/70 (Fig 5H) and 16/70 (Fig 5I) (2Hz and 20Hz respectively) showed a significant difference between CC_odour_ and CC_blank_. Further only 1/20 cell-odour pair among the ones showing significant change in the 2Hz case showed a CC_blank_ higher than the CC_odour_. This might be due to the residual respiration coupling left for this particular case.

To avoid the issue of the residual respiration driven membrane potential coupling contaminating the stimulus driven coupling, we computed a coupling coefficient index (C*p*C, Fig 5 A-F) for each individual cell (n = 42 cells, 70 cell-odour pairs, 25 mice, see Methods for details). In brief, to calculate C*p*C, the membrane potential was first baseline subtracted to minimise sniff-related membrane potential oscillations (Abraham et al., 2010) (Fig 5G). For a given cell-odour pair, C*p*C was then obtained by normalising the peak cross-correlation value for the odour response to that for the mineral-oil response (Fig 5 C-F), resulting in a C*p*C value > 0. A high C*p*C value indicates a cell that has a strong cell-odour frequency coupling relative to the baseline. Further, a C*p*C > 1 suggests a cell's response to the odour-frequency pair is stronger than that to a blank trial and is not due to a response of a potential residual purely mechanical stimulus. A subset of the recorded cell-odour pairs showed C*p*C >1 suggesting possible coupling to both 2Hz (35/70) (Fig 5J) and 20Hz (25/70) (Fig 5N). To assess statistical significance of this coupling measure, we estimated the C*p*C of all the baseline periods for a given cell and compared with the ones obtained for the odour period for all the trials (Fig 5K&O). Comparing the C*p*C between the original and the baseline, we observed that a substantial number of M/T cell-odour pairs indeed significantly coupled to both 2Hz (29/70 cell-odour pairs) (Fig 5L) and 20Hz (24/70 cell-odour pairs) (Fig 5P) (P<0.05, Two-tailed unpaired t-test; all of these were cell-odour pairs where we had observed significant subthreshold odour-evoked responses as defined above). However, we observed that 2/29 and 1/24 (2Hz & 20Hz respectively) cell-odour pairs among the significantly coupled ones showed a decrease in C*p*C for the odour stimulus compared to the baseline condition. For a subset of recorded cells, we presented a third stimulus, a continuous odour (with no temporal structure) and estimated the C*p*C as before. As expected, C*p*Cs obtained from the 2Hz and 20Hz stimuli was significantly higher than that from the constant odour stimulus for a substantial portion of the recorded M/T cells (2Hz: 17/32 cell-odour pairs; 20 Hz: 13/26 cell-odour pairs; P<0.01, Two-tailed paired t-test, Fig 5M&Q).

### Depth of recording correlated with C*p*C

**Fig 6:**
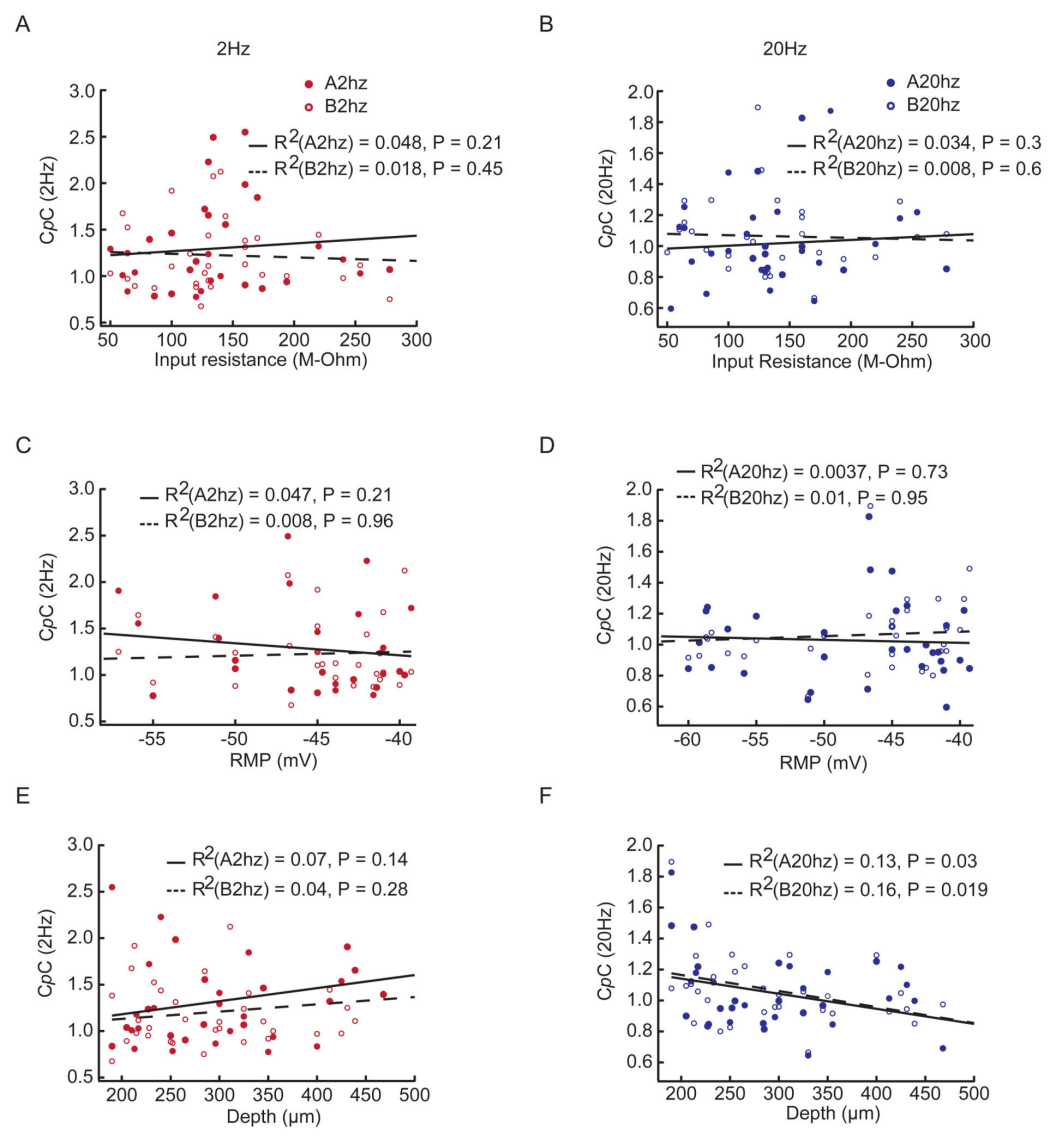
Cellular intrinsic properties do not explain the C*p*C of a cell. **(A)** C*p*C vs input resistance for the 2Hz and **(B)** 20Hz case. **(C)** C*p*C vs RMP for the 2Hz and **(D)** 20Hz case **(E)** C*p*C vs recording depth from the OB surface for the 2Hz and **(F)** 20Hz case. Note with increasing depth of recording estimated C*p*C significantly decreased for the 20Hz cases indicating TCs couple more to 20Hz odour stimulation than MCs.

We next asked if the C*p*C was related to the intrinsic properties of the recorded cells. We observed that input resistance (Fig 6A-B) and resting membrane potential (RMP, Fig 6C-D) of a cell were not correlated with its C*p*C. C*p*C and depth showed different correlations for 2Hz and 20Hz. For the 2Hz cases we could not find any correlation between depth and C*p*C (Fig 6E), while C*p*C decreased with depth for the 20Hz cases (Fig 6F) (P = 0.03, odour A and P = 0.019, odour B; Two-Tailed test). This suggests that tufted cells which are located more superficially than mitral cells might couple more strongly to high frequency (20 Hz) odour stimuli. Overall, however, C*p*C was only weakly dependent on intrinsic cellular properties.

### Putative Tufted cells show higher C*p*C than putative Mitral cells

**Fig 7:**
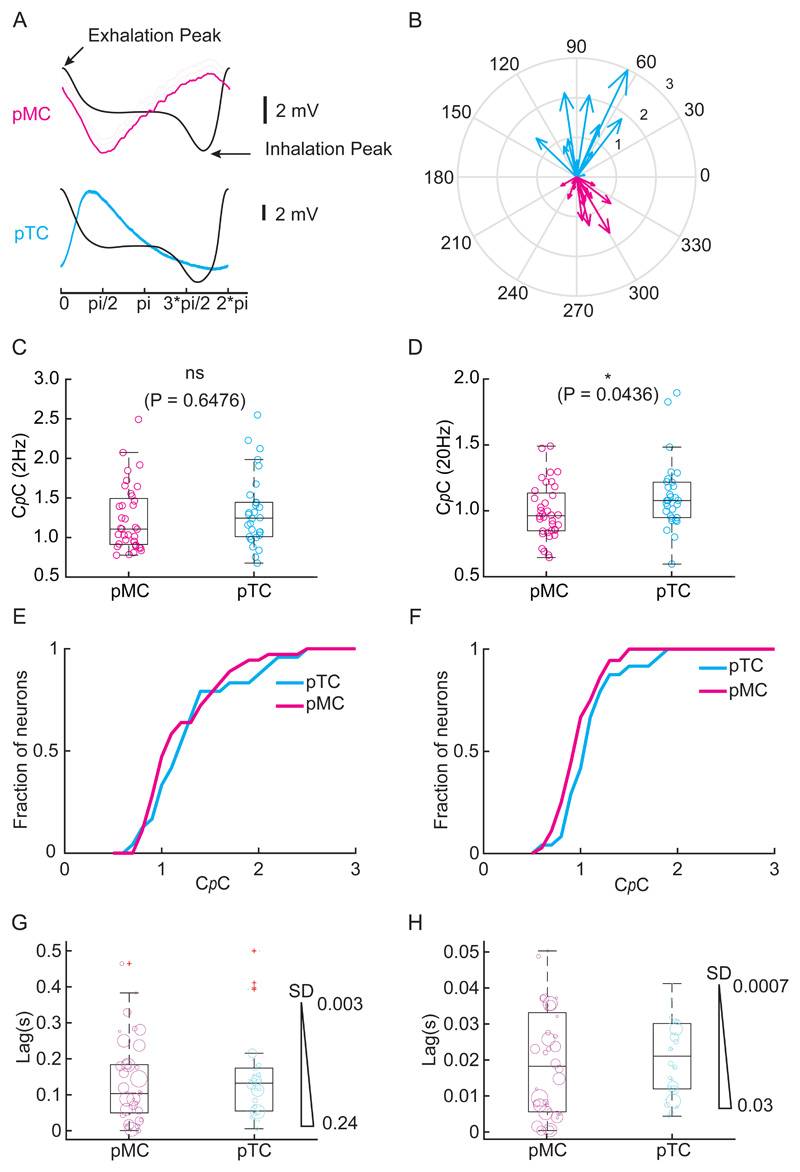
Putative Tufted cells show higher probability of following 20Hz stimulus but not 2Hz stimulus compared to putative Mitral cells. **(A) (*Top*)** Example baseline average V_m_ trace from a putative mitral cell (magenta) overlaid with average sniff cycle (black). ***(Bottom)*** similar traces for a putative tufted cell (cyan). **(B)** Summary phase diagram of peak subthreshold oscillation phase plot (pTC; n=17, pMC; n=23). **(C)** C*p*C for all the recoded pTC and pMC for 2Hz showing no significant difference between the two population (P=0.64, KS test) while **(D)** pTCs show higher C*p*C than pMCs for 20Hz cases (P=0.04, KS test). **(E)** Cumulative histogram of C*p*C for all pTC and pMC for 2Hz and **(F)** 20Hz cases. **(G)** Lag of the peak correlation point for the 2Hz case. The cyan and the magenta open circles represent individual pTC-odour pairs and pMC-odour pairs respectively. The population did not show any significant difference (P = 0.6297, Unpaired t Test). The marker size denotes the SD obtained from all the trials for a given cell **(H)** Same as in (G) but for the 20Hz case (P = 0.6634, Unpaired t Test).

Spontaneous oscillation of membrane potential has been observed to be a reliable predictor of projection neuron type in the OB (Fukunaga et al., 2012; Jordan et al., 2018b; Ackels et al., 2020). We classified the recorded neurons into 23 putative mitral cells (pMC) and 17 putative tufted cells (pTC) based on the phase locking of the spontaneous membrane potential to the respiration cycle of the mouse (Fig 7A-B). Two of the cells could not be resolved. While overall similar, pTCs tended to couple marginally more strongly to the odour stimuli than pMCs, reaching significance for the 20 Hz case (2 Hz: C*p*C_pTC_=1.32±0.48 (mean±SD), C*p*C_pMC_=1.24±0.41; P=0.6476, KS test; 20 Hz: C*p*C_pTC_=1.12±0.29, C*p*C_pMC_=0.95±0.49; P = 0.0436, KS test). This was consistent with the depth-based classification where we observed that for the 20Hz cases, superficially located (putative tufted) cells showed higher C*p*C compared to the deeper cells (putative mitral cells; Fig 6 F). However, we did not observe any significant difference in the lag of the peak correlation point between pTC and pMC (P = 0.6297, 2Hz; P = 0.6634, 20Hz, Unpaired t test). (Fig 7 G-H).

### C*p*C for odour mixtures can be linearly predicted from that of individual constituents

**Fig 8:**
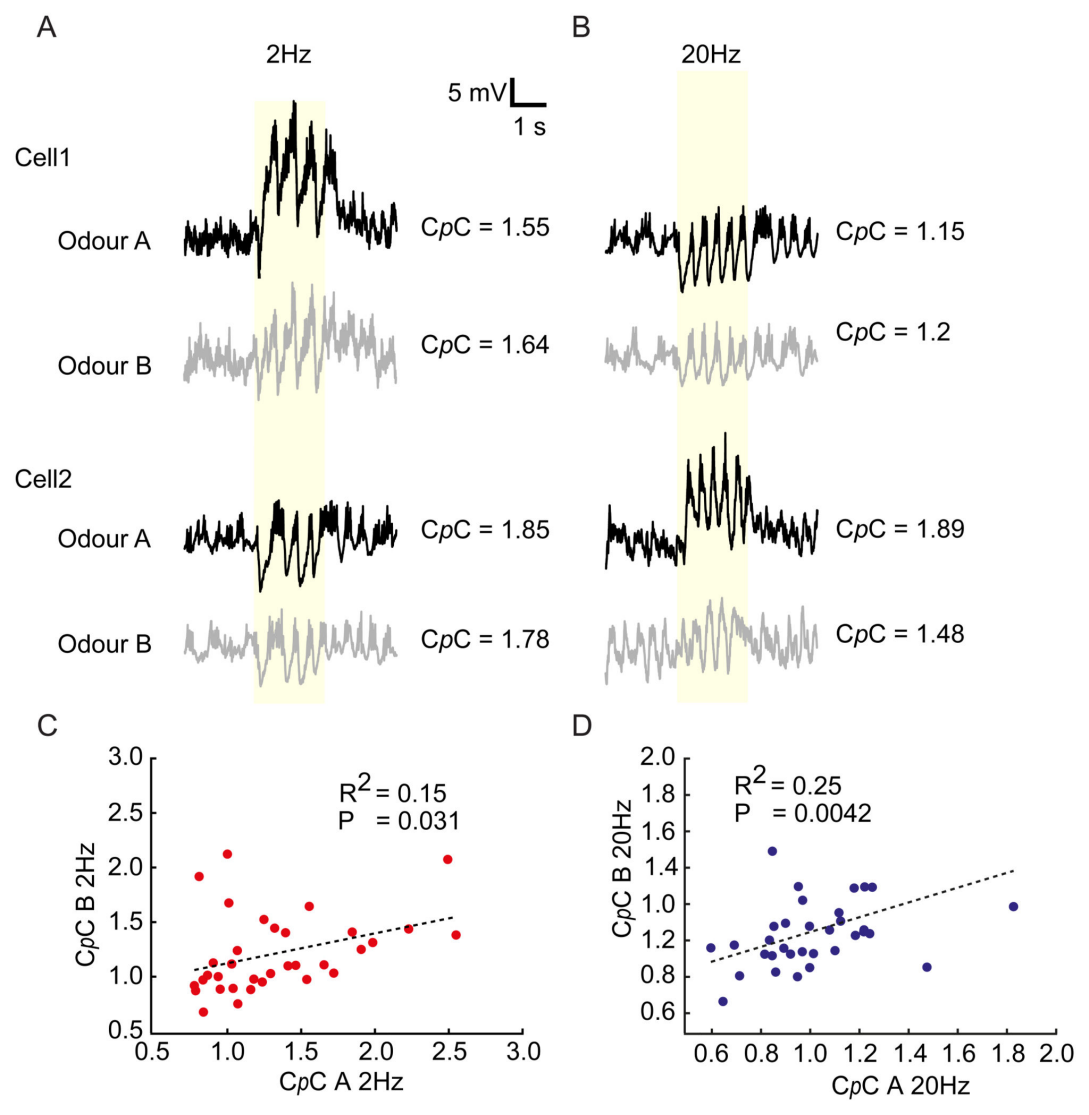
C*p*C is odour invariant. **(A)** Example recordings from two cells responding to both odours A and B at 2 Hz. Note that C*p*C values are similar for the two odours. **(B)** As in (A) for 20Hz stimulation. **(C)** Plot of estimated C*p*C A vs C*p*C B from all the recorded cells for 2Hz and **(D)** 20Hz case. Note the strong correlation in both cases suggesting that coupling to frequency-modulated odour stimuli is odour-independent.

**Fig 9:**
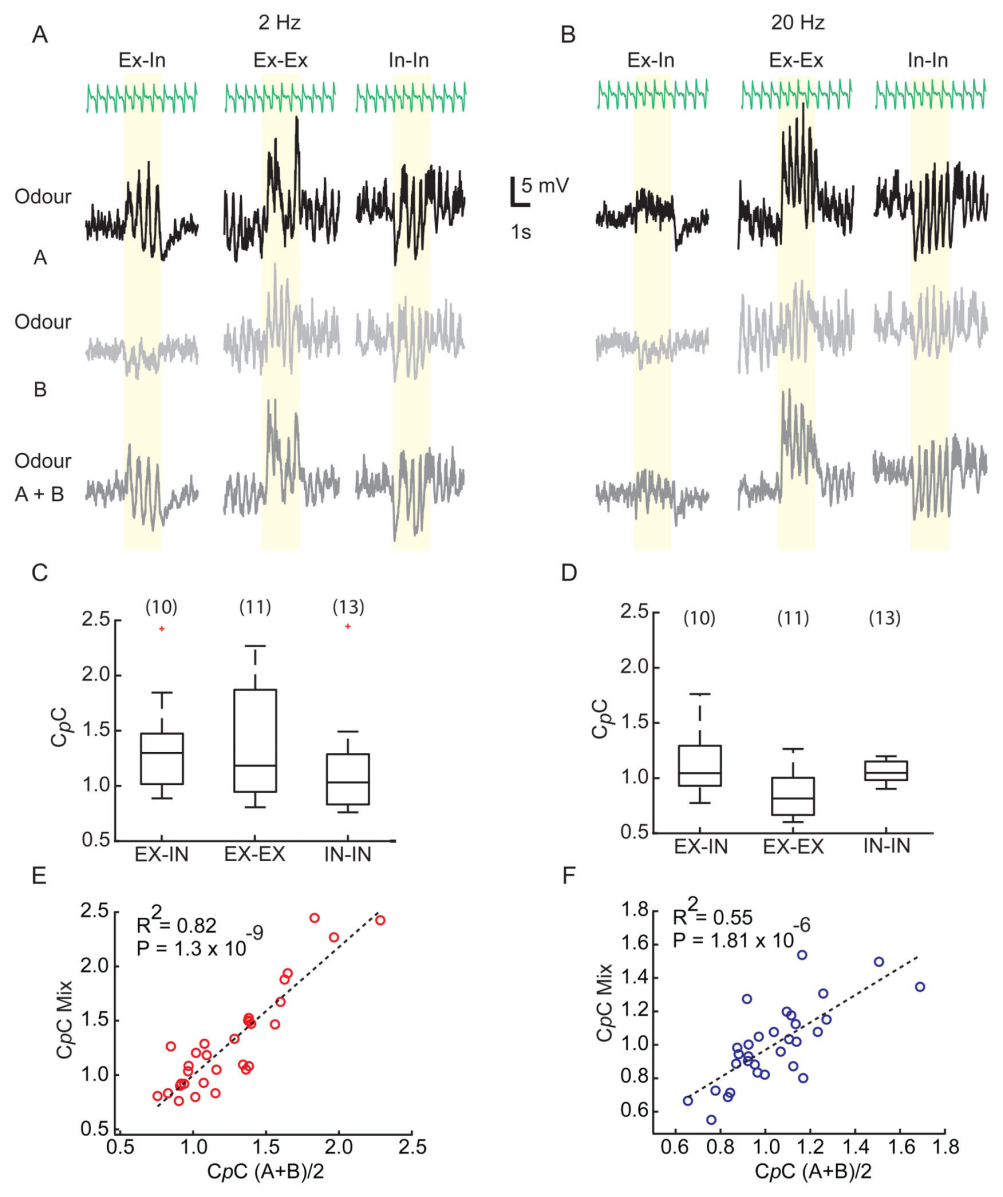
C*p*C for a mixture of odour can be linearly predicted from their individual component. **(A)** Example recordings for 3 different types of mixture interaction; excitatory-inhibitory (Ex-In), excitatory-excitatory (Ex-Ex) and inhibitory-inhibitory (In-In). From *top* to *bottom* respiration trace, V_m_ for odour A, B and A+B mixture delivered at 2Hz. **(B)** as (A) but for 20Hz. **(C)** No significant difference in C*p*C between the 3 types of mixture responses for 2Hz and **(D)** 20Hz. The numbers in bracket are the number of cells. (1-way Annova, P=0.54 (2Hz); P=0.14 (20Hz)) **(E)** Computed C*p*C of odour (A+B)/2 vs actual C*p*C of mixture (A+B) for 2Hz cases and **(F)** 20Hz cases. Note linear regression can reliably predict the relation between calculated and estimated C*p*C (n=35, P = 1.3x10^-9^, 2Hz; P = 1.81x10^-6^, 20Hz).

As described above, we presented two different odour stimuli at different frequencies (odour A and odour B). Comparing C*p*Cs for odour A with that displayed for odour B we noticed that these were tightly linked: M/T cells coupling well to odour A, also coupled well to odour B whereas M/T cells poorly coupling to one also coupled weakly to the other (Fig 8A-B). This was the case for both 2Hz (Fig 8C) and 20Hz (Fig 8D) suggesting that the frequency coupling of a cell is independent of the odour presented. To further corroborate that C*p*C is an odour-independent parameter, we probed M/T cells with a mixture of the two odours. If frequency-coupling is indeed odour-independent, a cell's response to odour mixtures should be predictable from the C*p*C for the individual odour. To assess this, we first categorized recordings based on the direction of average change in membrane potential in the first 500ms after odour onset, and classified responses into 3 types: excitatory-inhibitory (Ex-In), excitatory-excitatory (Ex-Ex), and inhibitory-inhibitory (In-In) (Fig 9A-B). Notably, we did not observe any significant difference between C*p*Cs for the different response types – neither for 2Hz (P = 0.54, One-way ANOVA; Fig 9C) nor for 20Hz (P = 0.15, One-way ANOVA; Fig 9D).

Next, we tried to predict the C*p*C of a given cell to an odour mixture, based on the cell's C*p*C obtained from the constituent odours. As predicted from the observation that C*p*C is largely cell-intrinsic and odour-independent, we observed that the C*p*C for a given cell-mixture pair could be reliably predicted from the cell-constituent pairs both for 2Hz (P = 1.3x10^-9^, Two-Tailed test) (Fig 9E) and 20Hz (P = 1.81x10^-6^, Two-Tailed test) (Fig 9F). Notably, we observed that C*p*C was not correlated with the strength of odour response for a given cell-odour pair (data not shown).

### Influence of inhibition on C*p*C

Since our observations indicated that C*p*C is cell-intrinsic, independent of the odour presented (Figs 8-9) and was only weakly dependent on intrinsic cellular properties (Fig 6), we next asked whether a cell's C*p*C was shaped by circuit properties.

**Fig 10:**
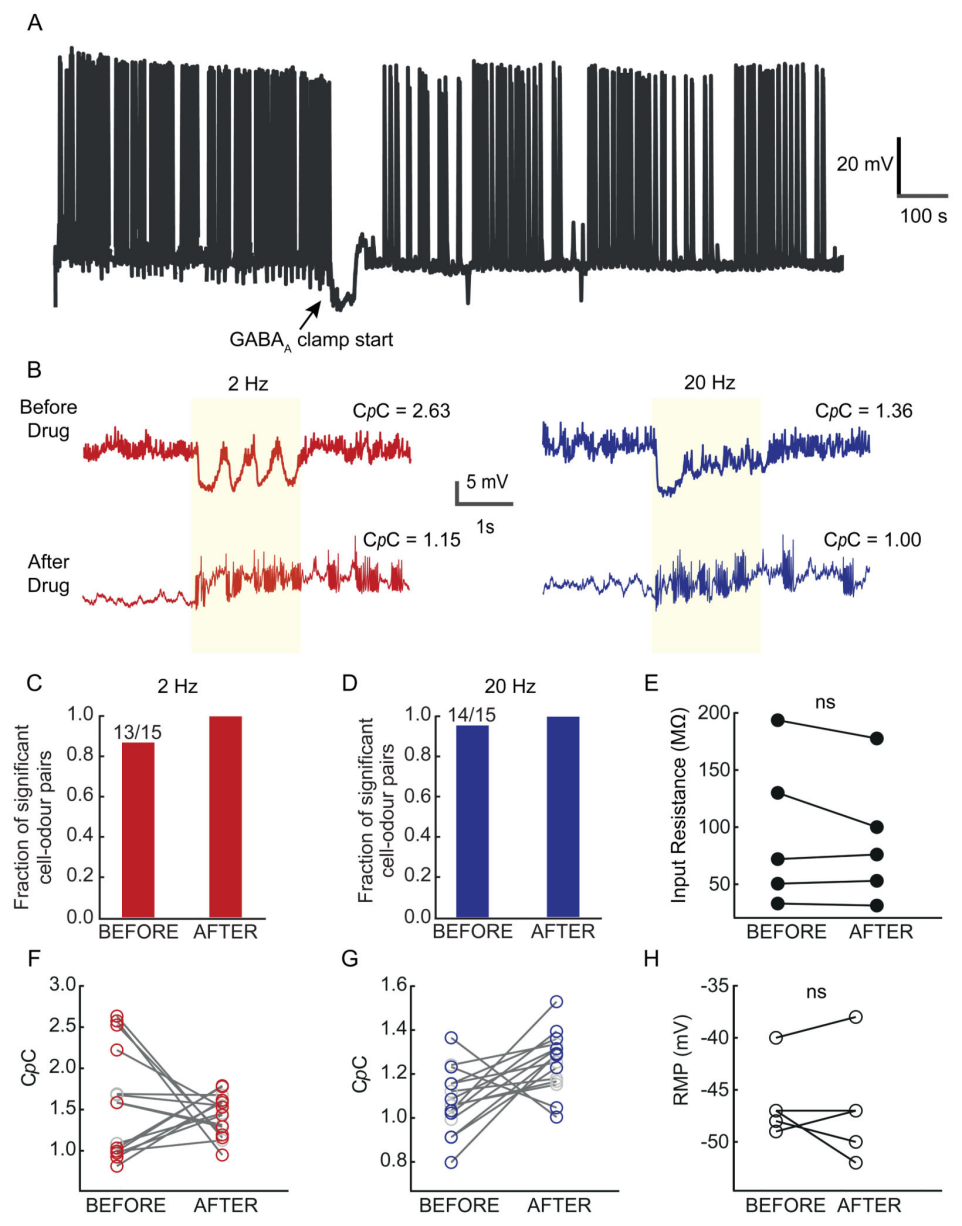
Influence of blocking inhibition on C*p*C. **(A)** Example recording showing time of 2mM Muscimol + 0.4mM Gabazine infusion for GABA_A_ clamping. **(B)** Example V_m_ trace without drug (***Top***) and after 10 minutes from drug infusion point **(*Bottom*)** in a continuous recording for 2Hz (red) and 20Hz case (blue). **(C)** Fraction of cell-odour pairs showing significant C*p*C compared to their baseline control before and after the drug infusion point for 2Hz stimuli. **(D)** Same as in (C) but for 20Hz cases. **(E)** Input resistance of the recorded neurons estimated both before and after drug infusion (n=5). No significant change was observed (Paired t test; P=0.272 (ns)). **(F)** 10/15 cell-odour pairs (red) showed significant change in their C*p*C post GABA_A_ clamping while 5/15 (grey) showed no significant change in the 2Hz case (P<0.01, Two-tailed paired t-test). **(G)** 9/15 cell-odour pairs (blue) showed significant change in their C*p*C post GABA_A_ clamping while 6/15 (grey) showed no significant change in the 20Hz case (P<0.01, Two-tailed paired t-test). **(H)** RMP of the recorded cells did not change significantly due to GABA_A_ clamping (Paired t test; P=0.313 (ns)).

OB inhibitory circuits are known to shape M/T cell activity and odour responses (Yokoi et al., 1995; Fukunaga et al., 2012; Fukunaga et al., 2014; Burton, 2017). To assess the role that circuit level inhibition may have on cellular C*p*C, we recorded from M/T cells as outlined previously, and then washed in a titrated mixture of 0.4mM gabazine and 2mM muscimol to cause "GABA_A_ clamping" (Fukunaga et al., 2012), blocking synaptic inhibition while providing sufficient unspecific, background inhibition to avoid epileptic discharge. Following a short period of change in membrane potential, the recorded neurons returned to approximately their original RMP within a few minutes (Fig 10A). The input resistance (Fig 10E) and RMP (Fig 10H) did not change significantly in any of the neurons recorded. Under baseline conditions before GABA-clamping, the estimated C*p*C was significant compared to their baseline control for most of the recorded cell-odour pairs (13/15; 2Hz and 14/15; 20Hz, P<0.01, Two-tailed paired t-test). Post drug perfusion (with GABAA-clamp) all recorded cells were significantly coupled (15/15; 2 & 20Hz, P<0.01, Two-tailed paired t-test) (Fig 10C-D). Furthermore, we noticed a significant change in C*p*C for most of the cell-odour pairs post drug treatment from their baseline values (n = 10/15; 2Hz and 9/15; 20Hz, P<0.01, Two-tailed paired t test) (Fig 10F-G). Interestingly, this shift in C*p*C post GABA_A_ clamping happened in both directions with 5/15 cell-odour pairs showing a significant *decrease* and 5/15 a significant *increase* in their C*p*C values in the 2Hz case (Fig 10F). For the 20Hz case, we observed that 2/15 cell-odour pairs showed a *decrease* while for 7/15 C*p*C values *increased* (Fig 10G). Furthermore, we observed that cell-odour pairs which showed a significant *increase* in C*p*C post GABA_A_ clamp from their baseline values, largely had initial C*p*C < 1 (2Hz: 0.947±0.076, 20Hz: 0.98±0.11 (mean±SD)), while for cell-odour pairs showing a decrease post GABA_A_ clamp had initial C*p*C > 1 (2Hz: 2.31±0.39, 20Hz: 1.3±0.07 (mean±SD)). Overall, our results indicate that the local inhibitory circuitry contributes significantly to determining a cell's C*p*C.

## Discussion

Mammalian olfaction research has largely used odour identity (chemical structure) and intensity as modulators of odour responses despite the presence of rich temporal structure in natural odour landscapes. Here we have shown that M/T cells in the OB *in vivo* can encode frequencies in odour stimuli as high as 20Hz. Furthermore, whole-cell recordings indicated that a subset of M/T cells significantly couple to frequencies of odour stimuli both at the sniff range and sub-sniff range in a largely odour-independent manner. Importantly, while heterogeneous between cells, the strength of coupling is largely independent of the odour applied. We have demonstrated that odour frequency coupling capacity is similar between pMCs and pTCs populations at 2Hz, while at 20 Hz, pTCs couple somewhat more strongly than pMCs. Finally, while coupling capacity is variable between cells but largely independent of specific intrinsic properties, we observed that inhibitory circuits strongly modulate a cell's frequency coupling capacity. Overall, we show that the OB has the capacity to encode high frequency temporal patterns present in olfactory stimuli. M/T cells vary in their propensity to follow temporally structured stimuli, and this depends on the local circuitry.

In mammals, respiration ensures a low-frequency, periodic sampling of olfactory stimuli which in turn is the main source of theta activity in the early olfactory areas (Macrides and Chorover, 1972; Margrie and Schaefer, 2003; Schaefer et al., 2006). This causes rhythmic activity in M/T cells, even when devoid of odour stimuli (Grosmaitre et al., 2007; Connelly et al., 2015; Diaz-Quesada et al., 2018). Furthermore, the concentration of natural odour stimuli fluctuates in time (Riffell et al., 2008; Martinez and Moraud, 2013; Celani et al., 2014; Pannunzi and Nowotny, 2019; Ackels et al., 2021) providing an extra layer of temporal information to the input signal of the OB. Altogether this creates temporally complex input signals for OSNs that are thought to carry information about the odour source (Hopfield, 1991; Vergassola et al., 2007; Celani et al., 2014; Ackels et al., 2021). While signal transduction at the olfactory receptor neuron level is relatively slow (Ghatpande and Reisert, 2011), simulations have shown that OSN convergence into the OB can help sustain high-frequency information (Ackels et al., 2021), similar to encoding in the auditory system (Carr, 1993). Furthermore, correlated and anti-correlated odour stimuli were shown to be faithfully represented in the OB and resulted in distinct behavioural responses for frequencies up to 40 Hz (Ackels et al., 2021).Together with the aforementioned physiological (Cury and Uchida, 2010; Shusterman et al., 2011) and behavioural experiments using precise optogenetic stimuli (Smear et al., 2011; Li et al., 2014; Rebello et al., 2014), this suggests the presence of cellular and/or network mechanisms supporting the encoding of high-frequency natural odour stimuli. Previous reports have varied inhalation frequency via tracheotomy and shown that M/T cells followed the respiratory rhythm at frequencies up to 5Hz (Diaz-Quesada et al., 2018; Short and Wachowiak, 2019; Eiting and Wachowiak, 2020). Here we have shown that in naturally breathing mice M/T cells in the OB can follow odour fluctuations well exceeding respiration rate at frequencies of 20Hz and that local circuit inhibition plays an important role in this encoding.

### Functional implications

Naturally odours are carried by turbulent plumes of wind or water generating filamentous fluctuations of odour concentration (Celani et al., 2014) that can in principle contain information about the nature and location of odour sources (Fackrell and Robins, 1982; Hopfield, 1991; Moore and Atema, 1991; Mylne and Mason, 1991; Vergassola et al., 2007; Schmuker et al., 2016; Ackels et al., 2021; Crimaldi et al., 2021; Marin et al., 2021). Our observations here indicate that the M/T cells reliably encode information about odour fluctuations at frequencies from 2-20 Hz (Fig 2-3 & 5). Consistent with these results obtained from unit recordings, we observe that indeed a subset of neurons recorded intracellularly showed significantly different spiking and subthreshold membrane potential activity when presented with 2Hz or 20Hz fluctuating odour stimuli (Fig 5K&O). Furthermore, our read-out parameter, *C*p*C,* provides an estimate of how strongly a given neuron can *directly* couple to a specific odour frequency. Both types of projection neurons coupled equally to odours presented at 2Hz (Fig 7C) while tufted cells showed somewhat higher coupling than mitral cells to 20Hz (Fig 6F&7D). It is possible that further physiological analysis with different temporally structured stimuli might reveal distinct subtypes of projection neurons. Recent reports have for example identified a subset of mitral/tufted cells specifically responsive to changes in concentration (Parabucki et al., 2019). Emerging molecular subtype division of projection neurons (Zeppilli et al., 2020) might reveal distinct groups of projection neurons following different temporal patterns of odour presentation. Considering the heterogeneity of projection targets, this might indicate that different postsynaptic regions receive differentially filtered information. Tufted cells that in our hands showed stronger frequency coupling for 20 Hz for example preferentially project to anterior olfactory nucleus or anterior piriform cortex (Scott et al., 1980; Schneider and Scott, 1983; Nagayama et al., 2010). Further studies will be required to find exact structures in the downstream areas related to frequency-specific odour signal computation. The fact that blocking inhibition altered the frequency coupling capacity (Fig 10) indicates that the inhibitory circuitry of the OB plays a strong role in shaping the encoding of temporal features. This is consistent with a previous finding in *Drosophila* projection neurons where blocking presynaptic inhibition altered response kinetics for temporally dynamic odour stimuli (Nagel et al., 2015). Additionally, we observe that post GABA_A_-clamping some of the cells displayed a decrease of C*p*C while other cells showed an increase (Fig 10F-G). Further studies are required to pinpoint the precise inhibitory pathway which might be responsible for modulating frequency-coupling in M/T cells in a cell-to-cell basis and possibly identify subpopulations of MCs and TCs that encode specific temporal features.

### Limitations of the study

All the recordings presented here were performed in anesthetized mice benefitting from the stability of respiration in this state. Previous reports suggested that the behavioural state affects mitral cell firing properties (Rinberg et al., 2006; Kato et al., 2012). However, studies have suggested that M/T cell firing rates in awake conditions do not change but rather get redistributed over a breathing cycle (Gschwend et al., 2012). Whole cell recordings from M/T cells indicate that membrane properties are largely similar between the two states (Kollo et al., 2014), consistent with recent unit recording results (Bolding et al., 2020). It will nevertheless be important to repeat these experiments in awake mice to probe if the M/T cells hold to the same frequency coupling behaviour as that found in anaesthetised. Secondly, owing to the time limitation of reliable whole-cell recordings we could probe only two frequencies. Extracellular unit recording data partially alleviated this limitation by investigating additional intermediate frequencies. Linear classifiers performed at accuracies well above chance level (Fig 3), suggesting that M/T cells can encode several frequencies up to 20Hz. Therefore, it is likely that the frequency coupling capacity of subthreshold activity could span this range as well.

### Temporally structured stimuli and pathophysiology

In addition to better replicating naturalistic stimuli, temporally patterned sensory stimuli have been found to be advantageous in treating diseases. While direct electrical (and more recently optical) rhythmic deep brain stimulation is recognised as a possible treatment for a variety of neurodegenerative diseases (Benabid et al., 1987; Laxton et al., 2010; Zhang et al., 2019), it is only quite recent that temporally modulated sensory stimulation has been employed to in a similar manner. For example using 40 Hz visual and/or auditory stimuli has been found to help alleviate the amyloid burden from medial prefrontal cortex (Martorell et al., 2019). These reports strongly suggest that temporally structured sensory stimuli can be used as a tool to treat AD patients. A recent report indicating that humans can use temporal olfactory cues suggests this may be possible for olfaction as well (Perl et al., 2020). Therefore, temporally structured odour stimuli could offer an additional route for treatment for neurodegenerative disorders.

Overall, in this study we report that M/T cells in the mouse olfactory bulb can encode temporal structure in odour stimuli and that their membrane potentials can follow frequencies of at least up to 20Hz. Extent of coupling is independent of the odours presented, varies between cells and is shaped by inhibition in the olfactory bulb.

## Significance Statement

Odours in the natural environment have a strong temporal structure which can be extracted and used by mice in their behaviour. Here, using *in vivo* extra- and intracellular electrophysiological techniques, we show that the projection neurons in the olfactory bulb can encode and couple to the dominant frequency present in an odour stimulus. Furthermore, frequency coupling was observed to be differential between mitral and tufted cells, was odour-invariant but strongly modulated by local inhibitory circuits. In summary, this study provides insight into how both cellular and circuit properties modulate encoding of odour temporal features in the mouse olfactory bulb.
